# The impact of internet use on health among older adults in China: a nationally representative study

**DOI:** 10.1186/s12889-024-18269-4

**Published:** 2024-04-17

**Authors:** Yanyan Zhou, Yang Bai, Jun Wang

**Affiliations:** 1 School of Agricultural Economics and Rural Development, Renmin University of China, Beijing, 100872 China; 2 School of Public Administration and Policy, Renmin University of China, Beijing, 100872 China; 3 Center for Health Policy Research and Evaluation, Renmin University of China, Beijing, 100872 China

**Keywords:** Older adults, Internet use, Health access, Lifestyle, Social interaction

## Abstract

**Background and objectives:**

Aging poses a significant challenge worldwide, with China’s aging status becoming particularly severe. What is the impact of Internet use on the health of the elderly? Existing studies have drawn conflicting conclusions that Internet use improves or harms health. The purpose of this study was to explore how Internet use affects the health of older adults, and the mechanisms and heterogeneity of these effects.

**Research design and methods:**

Based on Grossman’s theory, this paper constructs a health production function model. Using the data of China Family Panel Studies (CFPS) from 2014 to 2020, we investigated the impact of Internet use on the health of older adults using fixed effect model and instrumental variable method. We also examined the mediating role of health information acquisition, lifestyle, and social interaction in these associations.

**Results:**

Internet use is positively associated with self-rated health and negatively associated with psychological sub-health level. Internet use promotes the health of older adults by facilitating access to health information, healthy lifestyles, and enhancing social interaction. And the impacts are heterogeneous at the individual and regional levels.

**Discussion and implications:**

We should progressively enhance the level of internet accessibility for older adults, while concurrently addressing and narrowing the ‘digital divide’. By generating an abundance of superior health-related information, we can significantly improve health education tailored for the elderly. Additionally, it is crucial to offer extensive training opportunities designed to equip older adults with the necessary skills to proficiently navigate the internet.

**Supplementary Information:**

The online version contains supplementary material available at 10.1186/s12889-024-18269-4.

The health of older adults is important to Healthy China Strategy and also a major concern of whole world^1^. China’s aging situation is grim^2^. According to the National Bureau of Statistics, in 2022, the number of people aged 60 and above exceeded 280 million, accounting for 19.8% of the total population. In the context of deepening aging, the health of older adults^3^ has become particularly significant, and how healthy aging outperforms population aging is a major concern of the whole society.

One of the most important related research questions is: How does modern technology, especially the Internet, affect the health of older adults [1]? According to the 51st Statistical Report on Internet Development in China, as of December 2022, there were more than 152 million Internet users over 60 years old in China, accounting for 14.3% of the total number of Internet users.

In previous studies, there has been controversy about the impact of Internet use on the health of older adults. Using data from the Health and Retirement Study, Cotten [2] showed that internet use was associated with reduced depressive symptoms. Some scholars found that Internet use helps older people maintain a better level of physical and mental health, and better access to social support from family, friends, and neighbors, thereby improving their subjective well-being and promoting active aging [3]. Tang [4] analyzed the use of the Internet of older adults and found that using Internet information acquisition would lead to the shrinkage of their social network, thereby aggravating their loneliness. In addition, cross-sectional data was mostly used in previous studies, and causal effect analysis is needed.

In this paper, we addressed the above problems by using data of China Family Panel Studies (CFPS) from 2014 to 2020 to construct a fixed effects model and including the number of fixed telephones per 100 people in 1984 as an instrumental variable. And based on the Health Production Model, this paper deeply analyzes the mechanism of Internet use affecting the health of older adults.

## Literature review

In terms of how Internet use affects the health of older adults, there are mainly three views in existing research, including promotion theory, inhibition theory and non-impact theory.

The promotion theory holds that Internet use has a positive impact on the mental and physical health of older people. The use of the Internet is conducive to expanding their social connections and increasing their social capital. At the same time, Internet use plays an important role in alleviating loneliness and improving happiness, that is, the “network gain effect theory” [5–7]. In terms of physical health, the increase in age will decrease the cognitive ability of older adults, and the health status will gradually deteriorate. Older people can use the Internet to access health information and skills by actively integrating into digitalization to slow down the deterioration and even improve their health. Meischke [8] found that for older adults with hypertension and heart disease, searching and mastering relevant healthcare knowledge through the Internet is conducive to health management and effectively reducing morbidity. At the same time, Internet use can also improve the health of older adults by increasing their likelihood of exercise [9]. Cui et al. [10] found that the frequency of digital use significantly improves the diet, sleep, exercise, smoking, and alcohol consumption of Chinese older adults. Digital technology can promote healthy lifestyles in various ways, thereby improving the health status of Chinese older adults.

The impact of internet usage on the health of the elderly can be articulated through a refined understanding of three pivotal dimensions. Initially, the internet empowers seniors with “health knowledge.” Grounded in the Knowledge Attitude Practice (KAP) model, which posits a sequential influence from knowledge to attitude and finally to behavior, the internet emerges as a vital conduit for health information. It democratizes access to health knowledge, particularly benefiting older adults by broadening their understanding and enhancing their health management capabilities [11–13]. Research by Cohall et al. [14] underscores the potential of the internet to elevate the physical health of the elderly by facilitating the acquisition of pertinent health information.

Progressing from knowledge acquisition, the internet catalyzes the “practice” of healthy behaviors among the elderly. The Health Belief Model (HBM) elucidates this transition, highlighting the role of perceived threats, benefits, and barriers in shaping health-related behaviors [15]. The internet, by disseminating relevant information, aids older adults in recognizing the importance of healthy practices and overcoming obstacles to their adoption. This digital platform not only enhances awareness about the merits of physical activity and the perils of sedentary lifestyles but also fosters skill development for healthier living.

Furthermore, the internet stands as a pivotal tool for fostering “social interaction,” which is instrumental in the health and well-being of the elderly. The “Internet Gain Effect Theory” posits that the digital world offers new avenues for social engagement, thereby mitigating loneliness and depression, and bolstering mental and physical health [16]. Empirical studies, such as Boekel’s [17] investigation in the Netherlands, affirm the capacity of the internet to sustain and expand social networks for older adults, thereby enriching their social capital and access to information.

The second view is the inhibition theory. Although most studies have shown that Internet use can improve the health of older adults, it may also endanger their health due to excessive use or even addiction. Older adults who have insufficient ability to recognize Internet information, are easy to believe all kinds of false information, and even encounter fraud. At the same time, the use of Internet may cause irregular rest, crowd out sleep time, and make life disordered.

Another view is the non-impact theory. It believes that the Internet use reflects the impact of socioeconomic status on health, and Internet use itself does not directly affect the health of older people. Research by Gracia and Herrero [18] shows that Internet use is not a significant determinant of the health of the elderly once an individual’s socioeconomic status is taken into account. Dickinson [19] also found that Internet use had no significant effect on physical functioning in older adults.

Drawing upon the analysis of the existing body of literature, several key insights emerge. Initially, it is evident that the literature presents divergent conclusions regarding the impact of internet use on the health of older adults. This discrepancy can be attributed to multiple factors. One significant factor is the variance in data types across studies; while some research is grounded in experimental data, which may not be broadly representative, others may utilize observational data, leading to differences in interpretability and applicability of results. Moreover, the temporal context of data collection introduces another layer of complexity. Early research, conducted during periods of lower internet penetration and amongst a demographically narrow sample of older internet users—often characterized by higher socioeconomic status—may not accurately reflect the current reality where internet use among the elderly is more widespread and diverse. Additionally, the heterogeneity inherent within the older adult population itself is a critical element that contributes to inconsistent findings [20, 21]. The internet, as a multifaceted tool, offers a spectrum of uses from informational to recreational and social; however, its impact is contingent upon the manner of its utilization. While it can serve as a gateway to valuable resources and connections, its misuse or the encounter with misleading information can have detrimental effects. Thus, the relationship between internet use and the health outcomes of older adults is complex and individualized, necessitating a nuanced exploration of the various mechanisms at play. This analysis underscores the need for further research that addresses these variables and examines the multifaceted nature of internet use among the elderly.

Secondly, the extant scholarly discourse predominantly relies on cross-sectional studies, which markedly omits the nuanced insights that panel data could unveil regarding the evolving behaviors of individuals over time. This reliance on static data snapshots fails to capture the longitudinal dynamics essential for understanding the causal relationships and temporal changes in internet usage and health outcomes among the elderly. Thirdly, the theoretical underpinnings guiding much of the existing research often fall short of providing a robust and comprehensive analysis of the pathways through which internet usage influences health outcomes. This gap underscores the necessity for more sophisticated theoretical models that can elucidate the complex mechanisms at play. Moreover, analyses that probe into the heterogeneity of the internet’s impact on older adults’ health predominantly focus on the individual level, frequently overlooking the potential influence of regional and contextual factors that could significantly modulate this relationship. In an endeavor to surmount these limitations, the current paper introduces a health production model and harnesses panel data spanning from 2014 to 2020 from the China Family Panel Studies (CFPS). This approach, which transcends the limitations of single-year cross-sectional analyses, leverages a fixed-effect model in conjunction with an instrumental variable methodology. This methodological framework enables a more incisive investigation into how internet usage pertains to the self-rated health and psychological well-being of older adults, providing a more granular understanding of these dynamics over time.

## Theoretical models and research hypotheses

The factors affecting health are complex and diverse. The health ecological model provides a good perspective to analyze what factors affect the health of the elderly, and lays a foundation for the model construction and heterogeneity analysis of this paper [22]. This paper examines how Internet use affects the health of older adults from the second and third layers. In order to clarify the impact mechanism of Internet use on the health of older adults, this paper constructs a healthy production function model. It is based on the Grossman’theory [23], which has been widely used in various studies. For example, Zhang et al. [24] selected control variables based on the Grossman’theory. Aregbeshola & Khan [25] selected explanatory variables based on Grossman’s model.

Consider that individuals have the dual roles of consumer and producer. Assuming that at any given period $t$, as a consumer, personal utility is determined by health ${H}_{t}$ and non-health consumer goods ${Z}_{t}$, the individual’s utility in period $t$ is:

1 $${U}_{t}=U({H}_{t},{Z}_{t})$$

Faced with time constraints, an individual’s time $T$ is fully allocated to four activities, namely, ${T}^{W}$ Work: earning income to improve $H$ and $Z$; ${T}^{Z}$: Time spent to improve $Z$, such as leisure, travel; ${T}^{H}$: Time spent to enhance $H$, such as exercise; ${T}^{S}$: Time of illness, during which nothing can be produced and it is entirely determined by $H$. The constraint lines are:

2 $$T={T}^{W}+{T}^{Z}+{T}^{H}+{T}^{S}$$

As producers, individuals produce $H \,\text{a}\text{n}\text{d}\,Z$ that satisfy utility by combining the goods bought from the market with their own time. That is, the production of $H\, \text{a}\text{n}\text{d} \,Z$ requires two inputs: market goods and personal time. $M$ represents market goods that produce health $H$, such as fitness equipment, and $J$ represents market goods that produce non-health consumer goods $Z$, such as tickets. It is worth noting that $Z$ is a flow that is consumed in each period produced, while $H$ is the stock, similar to capital, which is accumulated and consumed in each period. Thus, at any given $t$ period, the production functions of ${H}_{t}\,\text{a}\text{n}\text{d} \,{Z}_{t}$ are:

3 $${H}_{t}=H({H}_{t-1},{T}_{t}^{H},{M}_{t})$$

4 $${Z}_{t}=Z({T}_{t}^{Z},{J}_{t})$$

Individuals face budget and time constraints. Assuming that in the period $t$, the remuneration per unit of work is $w$, then the total income is $Y_t=w\cdot T_t^W$, and the individual can allocate the income between the two types of goods whose prices are ${P}_{m} \,\text{a}\text{n}\text{d} \,{P}_{j}$ respectively, then the budget constraint is:

5 $$P_m\cdot M_t+P_j\cdot J_t\leq w\cdot T_t^W=Y_t$$

Assume that for older adults, ${T}^{W}\left(\text{w}\text{o}\text{r}\text{k}\text{i}\text{n}\text{g} \,\text{h}\text{o}\text{u}\text{r}\text{s}\right)=0$, and their income comes from pensions, child support, etc., and ${Y}_{t}$ is a fixed value. So, in the model for older people, there is no need to consider working hours. Production time ${T}^{P}$ is non-sick time, that is, the time used for the production of health and non-health consumer goods. In any given period, if the individual is healthier, then the duration of illness $({T}^{S})$ is shorter and the production time $({T}^{P})$ is longer. The production time constraint for older adults is:

6 $${T}^{P}=T-{T}^{S}={T}^{Z}+{T}^{H}$$

At the same time, the marginal return of health on productive time is diminishing, i.e. $\frac{\partial T^P}{\partial H}>0,\;\frac{\partial^2T^P}{\partial H^2}<0$. When a person is healthy enough, the increase in production time brought by an extra unit of health is small. When it is very unhealthy, the duration of illness is long, and even a small increase in health can bring a larger growth in production time. And there is a minimum point of health level, ${H}_{min}$, at which ${T}^{P}=0$, indicating that the entire period is sick, which is effectively equal to death. Based on the model, we propose three hypotheses:

- H1: Internet use improves the health of older people by facilitating access to health information.

Internet use facilitates access to health information for older people, so that they can acquire more health knowledge and improve their health production skills [26]. Search sites, chat groups, short videos and other platforms provide individuals with real-time, convenient, diverse and multi-point health information. It can help disseminate health information to older adults and enhance their healthcare capacity [13]. A study by Cotten [12] also found that people who used the Internet to search for health information had a higher level of health than those who searched by other means.

- H2: Internet use improves health by improving the lifestyle of older adults.

A good lifestyle is an important way to stay healthy [27]. The Internet’s publicity on healthy lifestyles such as strengthening exercise, health preservation, regular work and rest is conducive to the reasonable allocation of "production time". And the Internet has expanded the channels of sports publicity, which contributes to enhancing older adults’ awareness and skills, and even the frequency and intensity of exercise [28]. Exercise has a strong antidepressant effect, which is conducive to alleviating loneliness and improving happiness in older adults [29]. Therefore, the subtle impact of the Internet helps older adults improve their lifestyle, increase exercise time, and then enhance their health. So, this paper proposes:

- H3: Internet use improves the health of older adults by enhancing their social interaction.

Health ecology models show that multiple factors have an important impact on individual health. The new health production function is ${H}_{t}=AH\left({H}_{t-1},{T}_{t}^{H},{M}_{t},{X}_{t}\right)$ and ${X}_{t}$ denotes other factors affecting health, such as social interactions. The use of the Internet facilitates the connection and interaction between older adults and other people [30]. This helps maintain social relationships, stay active and socially engaged, overcome loneliness and depression, and receive physical and emotional health support [31], i.e., $\frac{\partial H}{\partial X_t}>0$.

## Data and methods

### Empirical strategy

In order to examine the impact of Internet use on the health of older adults, this paper constructs the following fixed effects model:

7 $${Y}_{it}={\beta }_{0}+\beta {frequency}_{it}+\gamma {X}_{it}+{county}_{i}+{year}_{t}+{\epsilon }_{it}$$

where $i$ represents older adults individual and $t$ represents the year. ${Y}_{it}$ is the level of health, including self-rated health (*health*) and mental sub-health (*poormental*); ${frequency}_{it}$ is the frequency of Internet use, and it is the independent variable we are interested in. ${X}_{it}$ is a series of control variables including individual and regional level, which will be detailed in the next “Variable definition” section. ${county}_{i}$ stands for county fixed effects, which allows this study to identify effects by within-district variation. It can absorb many geographic characteristics, especially the level of socioeconomic development, such as digital infrastructure and healthcare. ${year}_{t}$ is the year fixed effect, which helps to distinguish the time trend, such as the change of Internet penetration rate and health care policy. Standard errors are clustered at the provincial level. In order to ensure the robustness of our results, the clustering criteria will be changed subsequently and more stringent fixed effects will be added.

### Data

The data derives from the 2014–2020 dataset of the China Family Panel Studies (CFPS). CFPS covers 14,798 urban and rural households in 635 villages in 162 counties in 25 provinces in China. At the same time, the questionnaire conducted a comprehensive and detailed survey on the current situation of Internet use of older adults, as well as self-rated health and mental health. Based on the aim of our research, and according to the age classification criteria of the WHO, the sample was determined to be an elderly person who had reached 60 years old in 2014, and 23,679 valid samples were obtained after data processing.

### Measurements

The dependent variable is the health of older adults. We measure it through self-rated health and psychological sub-health. (1) Self-rated health (*health*), is measured by using the question “How do you think your health is?”, the answer is selected from “very healthy, healthy, relatively healthy, average, unhealthy”. They are expressed by a value of 5 − 1, the higher the value, the better the health. (2) Psychological sub-health (*poormental*), measured by 6 questions, that is “I feel depressed”, “I feel lonely”, “I feel sad”, “I feel that life cannot continue”, “I find it hard to do anything”, and “I don’t sleep well”. The answer is selected from “almost not, sometimes, often, most of the time.” The answer options are expressed by 1 − 4 respectively, and a higher value indicates a worse mental state.

The core independent variable is Internet usage, measured by Internet frequency (*frequency)*. It includes the use of the Internet for work, social, entertainment and other subdivision indicators. The answer options “almost daily, 3–4 times a week, 1–2 times a week, 2–3 times a month, once a month, once a few months, never” were assigned a value of 7 to 1, respectively. The *frequency* is the sum of the option scores, and the larger the value, the higher the frequency of Internet use.

The selection of control variables is based on the existing research, including individual and regional characteristics. Individual characteristics, including age(*age*), gender(*male*), live in rural or urban areas(*urban*), years of education(*edu*), whether have a spouse(*mate*), income(income), whether have medical insurance(insurance), whether to smoke(*smoke*) and nap(*nap*), and number of offspring(*offspring*). Because older adults with greater physical health may be more able to use the internet, we also control for "whether BMI is normal"(*BMI*), whether the body is uncomfortable(*discomfort*) and whether have diseases(*disease*) in order to alleviate endogenous problems. At the same time, this study controls for county fixed effects.

## Results

### Descriptive and regression results

Table 1 shows descriptive statistics for the variables. It shows that the average score of the self-rated health of older adults is 2.62, and the average score of psychological sub-health is 9.79, which means that their mental health is relatively good. Although the score for Internet use is increasing year by year, it is still at a low level. It can be observed that the health condition of the elderly is suboptimal, and their frequency of internet usage is low..

**Table 1:** Descriptive statistics

*Variables*	2014			2016			2018			2020		
	*N*	*mean*	*sd*	*N*	*mean*	*sd*	*N*	*mean*	*sd*	*N*	*mean*	*sd*
*health*	7,127	2.465	1.206	6,979	2.404	1.182	6,359	2.490	1.230	3,214	2.623	1.257
*poormental*	7,127	9.079	3.636	6,979	9.579	3.531	6,359	9.864	3.512	3,214	9.789	3.592
*frequency*	7,127	0.316	1.954	6,979	0.608	2.757	6,359	1.238	3.961	3,214	1.856	6.009
*urban*	7,127	0.485	0.500	6,979	0.480	0.500	6,359	0.479	0.500	3,214	0.470	0.499
*age*	7,127	67.98	6.769	6,979	68.42	6.708	6,359	68.73	6.114	3,214	68.91	5.677
*male*	7,127	0.516	0.500	6,979	0.514	0.500	6,359	0.519	0.500	3,214	0.517	0.500
*mate*	7,127	0.795	0.404	6,979	0.801	0.399	6,359	0.815	0.389	3,214	0.825	0.380
*insurance*	7,127	0.939	0.240	6,979	0.932	0.252	6,359	0.930	0.256	3,214	0.906	0.292
*income*	7,127	2,186	7,237	6,979	501.8	4,190	6,359	1,991	9,171	3,214	2,540	13,034
*edu*	7,127	4.211	4.489	6,979	4.386	4.533	6,359	1.971	1.117	3,214	5.272	4.691
*smoke*	7,127	0.159	0.366	6,979	0.159	0.365	6,359	0.178	0.383	3,214	0.157	0.364
*nap*	7,127	0.591	0.492	6,979	0.569	0.495	6,359	0.648	0.478	3,214	0.700	0.458
*BMI*	7,127	0.558	0.497	6,979	0.533	0.499	6,359	0.531	0.499	3,214	0.530	0.499
*offspring*	4,750	2.376	1.336	5,341	2.296	1.282	6,359	2.300	1.300	2,957	2.121	1.239
*discomfort*	7,127	0.411	0.492	6,979	0.404	0.491	6,359	0.423	0.494	3,214	0.357	0.479
*disease*	7,127	0.306	0.461	6,979	0.310	0.462	6,359	0.312	0.463	3,214	0.301	0.459

Table 2 reports the regression results. It shows that Internet use can promote the health of older adults significantly. Regardless of whether control variables were included, the coefficients of *frequency* were significant. For each unit increase in the frequency, the level of self-rated health increases by 0.006 units and psychological sub-health decreases by 0.019 units.

**Table 2:** Regression results

	(1)	(2)	(3)	(4)
*Variables*	*health*	*health*	*poormental*	*poormental*
*frequency*	0.016^***^	0.006^***^	-0.056^***^	-0.019^**^
	(0.002)	(0.002)	(0.009)	(0.008)
*Controls*	N	Y	N	Y
*FE*	Y	Y	Y	Y
*Observations*	23,673	19,403	23,673	19,403
*R-squared*	0.053	0.229	0.093	0.217

Table 2 reports the baseline OLS regression results from 2014 to 2020. The dependent variable in columns (1) and (2) is self-rated health (*health*), and columns (3) and (4) are mental sub-health level (*poormental*). The key independent variable is frequency of Internet use (*frequency*). All specifications include county and year fixed effects. Robust standard errors in parentheses are clustered at the provincial level

^*, **^ and ^***^ indicate significance at the 10%, 5%, and 1% levels, respectively

### Endogenous analysis

The accuracy of the benchmark regression results may be disturbed by reverse causation. The healthier older adults are, the more willing, energetic and able they are likely to use the Internet. In addition, another important source of endogenous problems is missing variables. Although we include the control variables covering the individual and regional levels, and control for the fixed effects of county and year, it may still miss some important variables that affect both the Internet use and the health of older adults. In order to address endogeneity problems, this paper uses the instrumental variable method, and we also introduce cross-fixed effect and random effect models.

By referencing previous studies, the number of fixed-line telephones per 100 people in each city in 1984 (phone) is used as the instrumental variable [32–39]. The reason why we choose this instrumental variable is that for the development of Internet technology in China, the first step is to access the Internet through fixed-line network, and then gradually develop to optical fiber broadband and wireless mobile phones. It can be inferred that the popularization of fixed-line telephones in history laid the foundation for the development of Internet technology. Therefore, the number of fixed-line telephones in cities in history meets the requirement of the instrumental variable’s correlation restriction. At the same time, compared with the rapid development of the Internet, the number of fixed-line telephones is unlikely to still directly affect the health of the elderly in the current era, thus satisfying the exclusion restriction of the instrumental variable. Table 3 reports the 2SLS regression results based on the instrumental variables, and the relevant test indicators of instrumental variables show that the selection is reasonable. Columns (1) and (2) of Table 3 show that the coefficient of the frequency is significant at the level of 1%, indicating that the benchmark regression conclusion is robust after the instrumental variable method alleviates the endogeneity problem. In addition, we also use the two-way fixed effects model and the random effects model. The results are shown in columns (3), (4), (5) and (6) of Table 3. After controlling for the cross fixed effects, the coefficient of frequency of Internet use is still significant, and the results of the random effect model are similar, which indicates that Internet use can indeed promote the health of older adults.

**Table 3:** Treatment of endogenous problems

	(1)	(2)	(3)	(4)	(5)	(6)
	*health*	*poormental*	*health*	*poormental*	*health*	*poormental*
	IV		county-year fixed effect		random fixed effect	
*frequency*	0.023^*^	-0.502^***^	0.005^***^	-0.018^**^	0.006^***^	-0.014^***^
	(0.012)	(0.048)	(0.001)	(0.008)	(0.002)	(0.005)
*Controls*	Y	Y	Y	Y	Y	Y
*FE*	Y	Y	Y	Y	Y	Y
*Kleibergen-Paap rk LM*	212.491^***^					
*Kleibergen-Paap rk Wald F*	251.948^***^					
*Observations*	23,679	23,679	19,307	19,307	19,407	19,407
*R-squared*	0.193	-0.053	0.250	0.249		

Table 3 reports the two-stage least squares regression results, the results of county-year cross fixed effects, and the results of random fixed effects of Internet use and elderly health and control variables, respectively. The dependent variable in columns (1), (3) and (5) is self-rated health (*health*), the dependent variable in columns (2), (4) and (6) is mental sub-health (*poormental*), and the key independent variable is the frequency of Internet use (*frequency*). Robust standard errors are clustered at the provincial level (robust standard errors in parentheses)

^*, **^ and ^***^ indicate significance at the 10%, 5%, and 1% levels, respectively

## Mechanisms and heterogeneity

### Test of mechanism

#### Health information access effect

Kenneth Arrow [40] pointed out that information asymmetry was a prominent feature of the medical service market. Especially among older adults, their ability to grasp information is insufficient, and there is an extreme lack of understanding of health information. Relevant studies have found that health information and related services provided by the Internet can make older adults pay more attention to their own health and have good health awareness, thus helping to improve their health [41]. We use the importance of using the Internet to obtain information (*info*) for older adults to measure the health information acquisition, and the following model is set:

8 $${info}_{it}={\beta }_{0}+\beta {frequency}_{it}+\gamma {X}_{it}+{county}_{i}+{year}_{t}+{\epsilon }_{it}$$

Column (1) of Table 4 presents the results of the test for the health information access effect. The coefficient for the frequency is significantly positive, indicating that Internet use improves the health level of older adults by promoting their access to health information, and Hypothesis 1 is verified.

**Table 4:** Mechanism analysis

	(1)	(2)	(3)	(4)	(5)	(6)
*Variables*	*info*	*info*	*exercise*	*exercise*	*social*	*recreation*
*frequency*	0.124^***^		0.035^**^		0.051^***^	0.046^***^
	(0.005)		(0.016)		(0.008)	(0.005)
*Internet*		1.726^***^		0.619^***^		
		(0.058)		(0.199)		
*Controls*	Y	Y	Y	Y	Y	Y
*FE*	Y	Y	Y	Y	Y	Y
*Observations*	19,403	19,399	19,403	19,399	2,120	2,120
*R-squared*	0.317	0.353	0.082	0.082	0.557	0.420

Table 4 reports the mechanistic test results for Internet use. The dependent variable in columns (1) and (2) is the importance of using the Internet to obtain information (*info*), which measures the effect of health information acquisition. It is based on the question “How important is the Internet to your access to information?” The scale ranges from 1 to 5, where 1 indicates “very unimportant,” 2 indicates “relatively unimportant,” 3 indicates “average,” 4 indicates “relatively important,” and 5 indicates “very important.” The dependent variable in columns (3) and (4) is the weekly exercise time(hours) (*exercise*), which measures the healthy lifestyle of older adults. The dependent variables in columns (5) and (6) are social and recreation (*social，recreation*), which measure the level of social interaction, and they are measured on a scale of “importance of social and entertainment when using the Internet” And 1 represents “very unimportant,” 2 represents “relatively unimportant,” 3 represents “average,” 4 represents “relatively important,” and 5 represents “very important.” The key independent variable in (1), (3), (5) and (6) is the frequency of Internet use (*frequency*). The key independent variable in (2) and (4) is whether to use the Internet (*Internet*). All specifications include county and year fixed effects. Robust standard errors are clustered at the provincial level (robust standard errors in parentheses)

^*, **^ and ^***^ indicate significance at the 10%, 5%, and 1% levels, respectively

#### Lifestyle improvement effect

The use of the Internet can help older adults recognize the importance of healthy lifestyle through various health education information and the display of other people’s lifestyles. The weekly exercise time (*exercise*) is used to measure the lifestyle of older adults, and the following model is set:

9 $${exerciset}_{it}={\beta }_{0}+\beta {frequency}_{it}+\gamma {X}_{it}+{county}_{i}+{year}_{t}+{\epsilon }_{it}$$

Column (2) of Table 4 reports the test results of the lifestyle improvement effect. It shows that Internet use has a positive impact on the health of older adults through the lifestyle improvement effect, so Hypothesis 2 is verified.

#### Social interaction enhancement effect

The theory of resocialization holds that older adults can relieve depression caused by retirement by actively participating in various social activities, maintaining and expanding social networks [42]. Relevant studies have also shown that the social participation and interaction of older adults are conducive to improving their mental health, alleviating loneliness and other negative emotions [31]. The level of social interaction is measured by social contact (*social*)and entertainment (*recreation*).

Internet use facilitates the contact between older adults and other people, which enhances the social interaction [30]. At the same time, short videos, live broadcasts, online mini games and other platforms that integrate entertainment and social interaction enrich their lives. So, Internet use can promote social interaction and thus improve the mental health of older adults, and Hypothesis 3 is verified.

### Heterogeneity analysis

We examine the heterogeneity of the impact of Internet use from multiple aspects through grouped regression, and the regression results can be seen in Tables 1 and 2 in the Appendix. Internet use affects older women to a greater extent than older men. When it comes to age, Internet use has no significant impact on the mental health of older adults aged 80–89. This may be due to the fact that there are obstacles for the very old to use the Internet, and the health effects of the Internet are limited for them. In addition, the urban-rural dual structure is a distinctive feature of China, there is a huge gap between urban and rural areas in terms of living conditions, income level, education level, pension and medical services. The degree of digitalization in rural areas is relatively low and the application of the Internet by the rural elderly is still superficial. As a result, the promotion effect of Internet use on their health lags behind that of urban elderly.

We also conducted grouped regression according to the four regions of East, Central, West and Northeast^4^. At the same time, Beijing, Tianjin, Shandong, Jiangsu, Shanghai, Zhejiang, Fujian, and Guangdong are considered as developed regions, and the remaining provinces are considered as non-developed regions for regression [43]^5^. In addition, this study constructs a regional Internet usage index (*userate*)^6^, which is high if it is higher than the average, otherwise it is low. The results show that the Internet use has a more significant and stronger promotional effect on the self-rated health of older adults in the eastern and northeastern regions, the developed regions and areas with high Internet usage index.

## Discussion and conclusions

Previous literature supports our findings that Internet use can promote the health of older adults. By searching for health knowledge on the Internet, older adults can improve their ability to understand and manage health conditions. So, when they interact with doctors, they feel more confident and empowered [44]. At the same time, the acquisition of health knowledge can enhance the elderly’s awareness of the importance of a healthy lifestyle, which is conducive to promoting the adoption of a healthy lifestyle [9], and can also slow down the speed of cognitive decline [45]. In addition, the Internet provides a convenient tool for the elderly to communicate with the outside world. Social media communication is related to higher levels of social support and social contact, which in turn is related to lower loneliness among the elderly [46]. We empirically investigate the impact of Internet use on the health level of the elderly using CFPS survey data from 2014 to 2020.

The policy implications of this paper are as follows. First, Internet use has a positive impact on the health of older people, so measures should be taken to enable more older people to use the Internet. It is necessary to pay special attention to bridging the “digital divide” among older adults. For example, strengthen the construction of Internet infrastructure, reduce tariffs, provide training for older adults on Internet use, and configure elderly models for various network applications. Second, older people should be trained to learn how to use the Internet to access information, and regulation of information providers must be strengthened. In terms of health information,use the Internet and other means to carry out health education for older adults, produce high-quality health information, improve their health literacy, so as to improve their lifestyle through the Internet’s subtle influence. At the same time, the supervision of online information should be strengthened, and bloggers with high-quality content related to the health of older adults should be encouraged. Third, there is great heterogeneity among different older adults, so it is important to provide personalized and differentiated online training and other assistance for older adults with different socioeconomic status and individual characteristics.

The marginal contributions of this paper are as follows. First, we alleviate the endogeneity problem in a variety of ways. In terms of data, we use the panel data of CFPS from 2014 to 2020, which will better solve the problem of missing variables, and provide individual dynamic behavioral information. In terms of method, this paper uses the fixed effect instrumental variable method for empirical test. Second, this paper makes up for the deficiency of previous research in theoretical construction. Based on Grossman’s theory, we construct a health production model, and deeply explore the mechanism of Internet use on the health of older adults. Third, heterogeneity analysis at the non-individual level was added, which expands the perspective of current heterogeneity research. It also provides more nuanced evidence for formulating policies to promote healthy aging.

We also have some limitations. Firstly, the mechanism analysis is only based on the model constructed by ourselves, and it is limited to only three aspects without considering other possible influencing mechanisms. Secondly, this paper does not analyze the possible negative impact of Internet use on the health of the elderly in detail. And how to maximize the positive effects of the Internet and minimize the negative effects requires further research. Thirdly, the promotional effect of the Internet on the health of the elderly is limited, and the excessive increase of usage may also inhibit their health. Therefore, it is necessary to explore how the Internet can be combined with other ways to promote the health of the elderly and how to make the elderly use the Internet reasonably. In addition, because we are using observational data, the results of the study should be treated with caution, even if multiple methods are used. And due to the limitations of the data, despite controlling for variables such as education, smoking, sleep, and region, we may still overlook the diversity within the older adult population in terms of digital literacy, health literacy, and varying access to technology.

### Supplementary Information

**Supplementary Material 1.**

## Data Availability

Data are available from the authors by request from the website (http://www.isss.pku.edu.cn/cfps/).

## Footnotes

1 According to WHO’s Decade of Action on Healthy Ageing 2020–2030, in 2020, more people over the age of 60 will outnumber children under the age of 5 globally; By 2030, the number of people aged 60 and above will reach 1.4 billion globally. At the same time, most older people live in developing countries.

2 By 2050, the proportion of China’s population aged 65 or older will surpass that of the United States, Britain and Germany, and close in on Japan, according to the Peking University-Lancet Major Report on Healthy Aging in China. China is likely to become one of the “oldest countries” in the world.

3 The general standard of population aging is the proportion of the elderly population. Generally speaking, the aging society means that the population aged 60 and above accounts for 10% of the total population. This paper adopts this standard and defines the elderly as the population aged 60 and above.

4 The regional division is based on the National Bureau of Statistics “East, West, Central and Northeast Region Classification Method”.

5 Referring to the research of Chen (2022), the evaluation index system of provincial economic development is formed from five aspects: structural level, economic benefit, economic scale, people’s living and openness level. The entropy method is used to calculate the comprehensive guidance of the economic development level of each province, and regions are divided into two types, that is developed and non-developed regions.

6 Regional Internet usage is the proportion of the province’s surveyed population that uses the Internet.
