# Supplementary material for: The impact of internet use on health among older adults in China: a nationally representative study
Source: BMC Public Health. 2024 Apr 17;24:1065. doi: 10.1186/s12889-024-18269-4 (PMC11022463; doi:10.1186/s12889-024-18269-4)
Supplement: Supplementary file 1 — Supplementary Material 1. [file 12889_2024_18269_MOESM1_ESM.pdf]

Appendix

Table 1 Heterogeneity analysis based on individual characteristics

	(1)	(2)	(3)	(4)	(5)	(6)	(7)	(8)	(9)	(10)
	male		female		60-69years old		70-79 years old		80-89 years old	
VARIABLES	*health*	*poormental*	*health*	*poormental*	*health*	*poormental*	*health*	*poormental*	*health*	*poormental*
frequency	0.003*	-0.010	0.011***	-0.032***	0.004*	-0.017*	0.014**	-0.033**	0.030***	-0.027
	(0.002)	(0.008)	(0.003)	(0.010)	(0.002)	(0.008)	(0.005)	(0.014)	(0.010)	(0.037)
Observations	10,140	10,140	9,260	9,260	13,080	13,080	5,403	5,403	899	899

	(11)	(12)	(13)	(14)	(15)	(16)	(17)	(18)	(19)	(20)
	urban		rural		0yuan		1-1000yuan		>1000yuan	
VARIABLES	*health*	*poormental*	*health*	*poormental*	*health*	*poormental*	*health*	*poormental*	*health*	*poormental*
frequency	0.007***	-0.022**	0.005	-0.010	0.006**	-0.015*	-0.110***	-0.170***	0.007	-0.017
	(0.002)	(0.009)	(0.005)	(0.017)	(0.002)	(0.008)	(0.037)	(0.060)	(0.004)	(0.015)
Observations	9,173	9,173	10,219	10,219	14,401	14,401	2,288	2,288	2,671	2,671

Table 1 reports the results of heterogeneity analysis at the individual level. Columns (1) - (4) report the results of regression grouped by gender. Columns (5) - (10) report the regression results by age group. Columns (11) - (14) report the regression results according to urban and rural groups. Columns (15) - (20) report the results of the regression by income group. Columns (21) - (30) report the regression results by educational level. All specifications include county and year fixed effects. Robust standard errors are clustered at the provincial level (robust standard errors in parentheses). *, **, and *** indicate significance at the 10%, 5%, and 1% levels, respectively.

Table 2 Analysis of heterogeneity based on regional level

	(1)	(2)	(3)	(4)	(5)	(6)	(7)	(8)
	East		Northeast		Central		West	
VARIABLES	*health*	*poormental*	*health*	*poormental*	*health*	*poormental*	*health*	*poormental*
frequency	0.008***	-0.019	0.009***	-0.008	0.001	-0.029	0.005	-0.026
	(0.002)	(0.013)	(0.000)	(0.011)	(0.006)	(0.017)	(0.003)	(0.015)
Observations	7,064	7,064	2,958	2,958	4,762	4,762	4,594	4,594
	(9)	(10)	(11)	(12)	(13)	(14)	(15)	(16)
	non-developed regions		developed regions		High userate		Low userate	
VARIABLES	*health*	*poormental*	*health*	*poormental*	*health*	*poormental*	*health*	*poormental*
frequency	0.004**	-0.017*	0.014***	-0.028	0.007**	-0.027**	0.006**	-0.016
	(0.002)	(0.008)	(0.002)	(0.017)	(0.003)	(0.011)	(0.002)	(0.010)
Observations	14,950	14,950	4,436	4,436	10,897	10,897	8,487	8,487
Controls	Y	Y	Y	Y	Y	Y	Y	Y
FE	Y	Y	Y	Y	Y	Y	Y	Y

Table 2 reports the results of heterogeneity analysis at the regional level. Columns (1) - (8) report the regression results by group according to the eastern, central, western and northeastern regions. Columns (9) - (12) report the regression results by groups according to developed regions and non-developed regions. Columns (13) - (16) report the results of the regression by regional Internet usage (*userate*). All specifications include county and year fixed effects. Robust standard errors are clustered at the provincial level (robust standard errors in parentheses). *, **, and *** indicate significance at the 10%, 5%, and 1% levels, respectively.
